# The Use of ^**18**^F-FDG-PET/CT for Diagnosis and Treatment Monitoring of Inflammatory and Infectious Diseases

**DOI:** 10.1155/2013/623036

**Published:** 2013-08-21

**Authors:** Andor W. J. M. Glaudemans, Erik F. J. de Vries, Filippo Galli, Rudi A. J. O. Dierckx, Riemer H. J. A. Slart, Alberto Signore

**Affiliations:** ^1^Department of Nuclear Medicine and Molecular Imaging, University of Groningen, University Medical Center Groningen, Hanzeplein 1, 9700 RB Groningen, The Netherlands; ^2^Nuclear Medicine Unit, Department of Medical-Surgical Sciences and of Translational Medicine, Faculty of Medicine and Psychology, “Sapienza” University, Via di Grottarossa 1035, 00189 Rome, Italy

## Abstract

FDG-PET, combined with CT, is nowadays getting more and more relevant for the diagnosis of several infectious and inflammatory diseases and particularly for therapy monitoring. Thus, this paper gives special attention to the role of FDG-PET/CT in the diagnosis and therapy monitoring of infectious and inflammatory diseases. Enough evidence in the literature already exists about the usefulness of FDG-PET/CT in the diagnosis, management, and followup of patients with sarcoidosis, spondylodiscitis, and vasculitis. For other diseases, such as inflammatory bowel diseases, rheumatoid arthritis, autoimmune pancreatitis, and fungal infections, hard evidence is lacking, but studies also point out that FDG-PET/CT could be useful. It is of invaluable importance to have large prospective multicenter studies in this field to provide clear answers, not only for the status of nuclear medicine in general but also to reduce high costs of treatment.

## 1. Introduction

In recent years, the use of nuclear medicine to characterize and diagnose infectious and inflammatory diseases is rapidly increasing. Several SPECT and PET radiopharmaceuticals have been developed and applied in this field, radiolabelled white blood cells being the centerpiece [1]. However, [^18^F]-FDG-PET combined with low dose or diagnostic computed tomography (CT) is gaining interest in the diagnosis of many infectious and inflammatory diseases and is already the gold standard for some indications. 

The accumulation of FDG in inflammatory and infectious diseases is based on the high uptake in activated granulocytes. This accumulation is based on the fact that these cells use glucose as an energy source only after activation during the metabolic burst. Transport of FDG across the cellular membrane is mediated by the glucose transporter (GLUT) proteins, which are also to a higher amount present on the cell membrane of inflammatory and infectious cells [2].

For therapy followup, the indication of FDG-PET is less clear. The European Medicines Agency (EMEA) Committee for Medicinal Products for Human Use (CHMP) only mentions the use of FDG for the therapeutic followup of unresectable alveolar echinococcosis, in which it may be used in the search for active localizations of the parasite during medical treatment and after treatment discontinuation [3]. Despite this lack of attention, we think that FDG-PET/CT is not only valuable for therapy monitoring in some infectious and inflammatory diseases but could even play a pivotal role in their management, leading to better drug dosage, proof of the usefulness of the treatment, and early modification of the therapeutic strategy.

The literature for this review was collected with PubMed and Cochrane search using the combination of “FDG,” “therapy,” “therapy evaluation,” and the specific term for each inflammatory or infectious disease. The reference lists of selected articles were checked for additional valuable literature. This paper summarizes most papers published so far in this field and tries to define the role of FDG-PET/CT for therapy monitoring in various infectious and inflammatory diseases.

## 2. Vasculitis

### 2.1. FDG-PET/CT and Classification of Vasculitis

Systemic vasculitis is a multisystem disease characterized by inflammation with infiltration of leukocytes into the blood vessels. Classification of vasculitis is still unsatisfactory and controversial. Existing classifications—the American College of Rheumatology criteria [4, 5], the Chapel Hill Consensus Conference definitions [6], and the adapted Zeek classification system [7]—are useful but limited [8]. For nuclear medicine, the Zeek system is most useful since it reflects dominant vessel size, in association with antineutrophil cytoplasmic antibodies (ANCAs). 

For diagnosis, CT is useful for showing mural changes, including wall thickening, calcification, and mural thrombi. CT angiography (CTA) provides the possibility to reveal luminal changes, such as stenosis, occlusion, dilatation, and aneurysms [9]. MRI is probably the best method to evaluate and reveal structural vascular abnormalities (aneurysms, stenoses) but does not identify inflammation in structural normal blood vessels [10]. Because of the limited spatial resolution of the PET/CT camera, nuclear medicine is most of the times able to visualize only inflammation of the aorta and the larger arteries. However, depending on the spatial resolution of the used camera, it is possible to find FDG uptake also in smaller arteries, as other studies mentioned [11–13]. Furthermore, FDG-PET/CT may be able to differentiate between giant cell arteritis (GCA), Takayasu's arteritis (TA), and polyarteritis nodosa (PAN).

**Figure 1:**
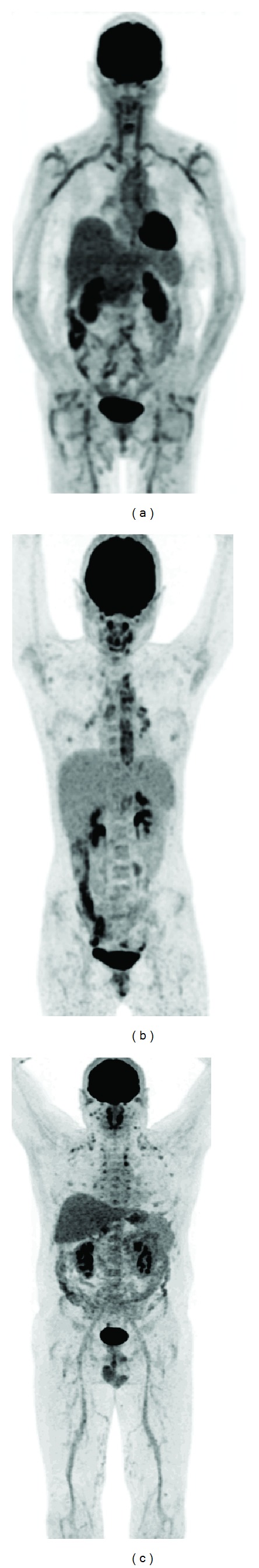
FDG-PET examples of vasculitis: (a) giant cell arteritis and polymyalgia rheumatica: high FDG uptake in the large vessels (aorta, subclavian arteries, carotid arteries, iliac arteries, and femoral arteries) accompanied by high uptake in the large joints (shoulders and hips), (b) Takayasu's arteritis: high FDG uptake located more centrally (aorta and main branches in the thoracic region) and in this case uptake in reactive lymph nodes in mediastinum and hili (confirmed by biopsy), and (c) polyarteritis nodosa and polychondritis: high uptake in the medium- and small-sized arteries (best visible in the legs) accompanied by uptake in the nose, the ears, and the costochondral regions.

GCA, also called temporal arteritis, is a granulomatous inflammation of the aorta and its main branches, most of the times occurring in patients older than 50 years. The extracranial branches are also involved, especially the temporal artery. Normally, involvement of the temporal artery is difficult to see on FDG-PET due to its small diameter, but involvement of the aorta, subclavian, carotid, and iliac arteries is enough to settle the diagnosis GCA based on FDG-PET findings. GCA is often associated with polymyalgia rheumatica (PMR), an inflammatory disease around the joints, causing pain and stiffness (Figure 1(a)).

Takayasu's arteritis is more centrally located (therefore also known as aortic arch syndrome) and mainly affects the aorta and the main branches in the thoracic region (carotid arteries, brachiocephalic trunk, subclavian arteries, and the pulmonary arteries). In the majority, it affects young or middle-aged woman. An example is shown in Figure 1(b). In this 14-year-old girl, Takayasu's arteritis was confirmed by biopsy, together with inflammatory lymph nodes in the mediastinum around the inflamed arteries.

PAN is a vasculitis of the medium and small-sized arteries, which become swollen and damaged by immune cells. Most cases occur at middle age, but it can affect nearly everyone. PAN may be associated with polychondritis [14], as shown in Figure 1(c). On FDG-PET, uptake may be seen in the smaller arteries, best visible in the extremities.

When choosing between FDG-PET/CT and MRI, it is important to consider both study-specific and patient-specific factors. For FDG-PET/CT, some questions still remain to be answered, for example, the usefulness of this technique in patients taking corticosteroids and immunosuppressives [15].

### 2.2. The Role of FDG-PET/CT in Diagnosing and Evaluating Therapy in Vasculitis

In the setting of diagnosing vasculitis, FDG-PET/CT was proven valid and utility of FDG-PET/CT was found in:

- the initial diagnosis of patients suspected of having a vasculitis, and particularly in patients that presented with nonspecific symptoms. In GCA, sensitivities ranging from 77 to 92% and specificities from 89 to 100% were reported [16]. In TA, results were even better (sensitivity 92%, specificity 100%) [17];
- the identification of areas of increased FDG uptake as a target site in which a biopsy should best be taken to obtain a definite diagnosis [18, 19];
- the evaluation of the extent of the disease and involvement of extracranial sites, with more vascular involvement found by FDG-PET/CT compared to MRI imaging and traditional angiography [20, 21]. A correlation was also found with inflammatory markers as C-reactive protein and erythrocyte sedimentation rate [21].

Many case reports are published that mention a role of FDG-PET/CT in guiding treatment strategy and evaluating therapy response. However, only a small number of large patient studies exist.

Blockmans et al. performed FDG-PET at baseline and after 3 and 6 months of therapy with corticosteroids in 35 patients with suspected GCA. The FDG-PET at baseline was positive in 29 patients, leading to a sensitivity of 83%. The total vascular score ((TVS) a scoring system of 0 to 3 points in 7 vascular regions using the Meller visual grading scale) decreased from 7.9 at baseline to 2.4 after 3 months of therapy. No further decrease was found at 6 months. However, in long-term followup, 18 relapses were found, with the TVS at baseline of this group of relapsing patients not differing from the whole patient group. The authors concluded that FDG-PET is sensitive for detecting GCA and that therapy evaluation after 3 months is possible without added value of scanning on a later time point. Relapse of GCA could not be predicted by FDG-PET [22].

Bertagna et al. performed FDG-PET/CT before and at least 4 months after corticosteroid therapy in 9 patients with proven vasculitis. Eight patients became negative after therapy (aorta/liver ratio < 1 or SUVmax < 2), which was concordant with clinical and biochemical findings. One patient remained positive on FDG-PET, which was also confirmed clinically and biochemically. Using FDG-PET/CT, instead of FDG-PET alone, allowed the authors to precisely identify the anatomical sites of disease, which was found particularly useful after therapy to identify possible weak persistence of disease. They concluded FDG-PET/CT being a useful, accurate tool for establishing the diagnosis of large-vessel vasculitis, for evaluating disease extension, and for monitoring therapy in conjunction with clinical and biochemical findings [10].

More recently, Henes et al. retrospectively studied 10 patients with proven vasculitis that were treated with cyclophosphamide, because they were not responding to corticosteroids and/or had organ or limb threatening vascular stenosis. FDG-PET/CT was performed before and during (6 out of 10 cycles mostly) therapy and the visual grading score of Meller was used. Before therapy, the grading score was 3 in 8 patients and 2 in 2 patients. During therapy, all patients had a grading score equal to or lower than 2. Nine out of 10 patients showed clinically a complete remission after 10 cycles of cyclophosphamide. The authors concluded that cyclophosphamide was an effective therapy in patients and that FDG-PET/CT might serve in future as an additional tool to continue successful treatment or stop the unsuccessful [23].

FDG-PET/CT was also found to have impact on the clinical management in a significant proportion of 30 patients with suspected GCA, both in patients without and already with immunosuppressive medication. The addition of FDG-PET increased the number of indicated biopsies and changed treatment recommendation in 27% of patients [24].

## 3. Sarcoidosis

### 3.1. FDG-PET/CT and Classification of Sarcoidosis

Sarcoidosis is a multisystem granulomatous disease that affects predominantly the lungs and associated lymph nodes but may involve virtually any organ. Despite the progress in imaging techniques, only one imaging classification system exists that was already described more than 4 decades ago [25]. This system classifies chest radiographic findings as stage 0 (normal radiography), stage I (bilateral hilar lymphadenopathy), stage II (stage I and parenchymal infiltration), stage III (parenchymal infiltration without hilar lymphadenopathy), and stage IV (parenchymal infiltration with pulmonary fibrosis) [26]. Of course, this classification system is obsolete, because of the currently existing imaging modalities.

For conventional imaging, high resolution computed tomography (HRCT) is now widely accepted as the radiographic imaging reference standard in the evaluation of sarcoidosis and other diffuse infiltrative lung diseases [27]. HRCT is superior to conventional CT in delineating the distribution and pattern of pulmonary interstitial lesions [26] and has typical findings for sarcoidosis with small nodules in perilymphatic distribution or along fissures but also with alveolar consolidation with air bronchograms, cavitation, and fibrosis when there is lung involvement. Sensitivities reported for HRCT in diffuse infiltrative lung diseases are high (>90%) [28]; however, it is often difficult to differentiate sarcoidosis from other interstitial diseases, and biopsy is still required.

**Figure 2:**
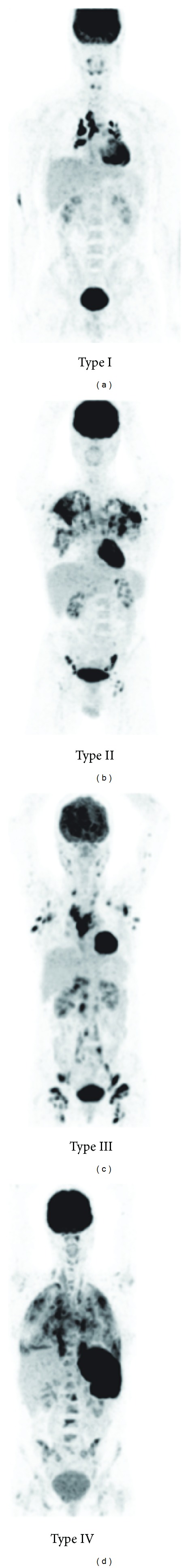
FDG-PET classification of sarcoidosis. Type I: thoracic lymph node involvement (in this image: mediastinal and hilar regions), type II: involvement of the lung parenchyma, type III: diffuse lymph node involvement (in this image all lymph node regions of the body are involved), and type IV: organ involvement (in this image involvement of the spleen and bones) (images courtesy by R. Keijsers).

FDG-PET provides valuable information in this disease as FDG is highly taken up by the granulomas. Moreover, whole body imaging is possible with FDG-PET. Different presentations of sarcoidosis on FDG-PET exist. Keijsers et al. categorized FDG-PET patterns in sarcoidosis patients based on the presence and extent of organ involvement. The involvement of thoracic lymph nodes and lung parenchyma was considered as the presence of extrathoracic disease (Figure 2). This system could have added value for prognosis and stratification as parenchymal disease, splenomegaly, and involvement of more than three organ systems is associated with a poor prognosis [29, 30]. 

For diagnosis, both FDG-PET and HRCT are necessary. HRCT is required because of its typical findings for sarcoidosis, as mentioned earlier, and because FDG-PET is not very specific: the uptake pattern in sarcoidosis can mimic malignancy or lymphoma disease. Despite this low specificity, FDG-PET has been extensively studied on a relatively large number of patients, and many publications support the use of FDG-PET for diagnosis, although in combination with (HR) CT.

FDG-PET has a high sensitivity for diagnosing sarcoidosis, and provides valuable information to evaluate pulmonary and extrapulmonary sarcoidosis [2]. Whole body FDG-PET imaging may uncover an occult diagnostic site or multiple organ involvement [31] and is also useful in cardiac [32] and cerebral [33] sarcoidosis. Compared with the old “gold standard” tracer for sarcoidosis, ^67^Ga-citrate SPECT, FDG-PET was found more suitable for imaging the mediastinum, the hilar lymph nodes, the posterior regions of the lungs, and nonthoracic lesions [34, 35]. The metabolic activity measured with FDG-PET reflects the disease activity in sarcoidosis in quantitative terms and the SUVmax correlates with histopathological results from bronchoalveolar lavage [36]. Diffuse parenchymal uptake of FDG predicts a future deterioration—when untreated—of the diffusion capacity of the lung and absence of activity in the lung parenchyma could justify a wait-and-see policy [37]. Furthermore, FDG-PET may show some specific features that could help in the diagnosis, for example, thick linear FDG uptake in the lower legs in muscular sarcoidosis (the so-called “tiger man” sign) [38].

### 3.2. FDG-PET/CT and Therapy Evaluation

For treatment of sarcoidosis, several options are possible: treatment with corticosteroids in various doses, antimalarial drugs such as hydroxychloroquine, cytotoxic drugs, and also with cytokine modulating drugs, such as infliximab or adalimumab, both being antitumor necrosis factor alpha (anti-TNF*α*) antibodies. 

**Figure 3:**
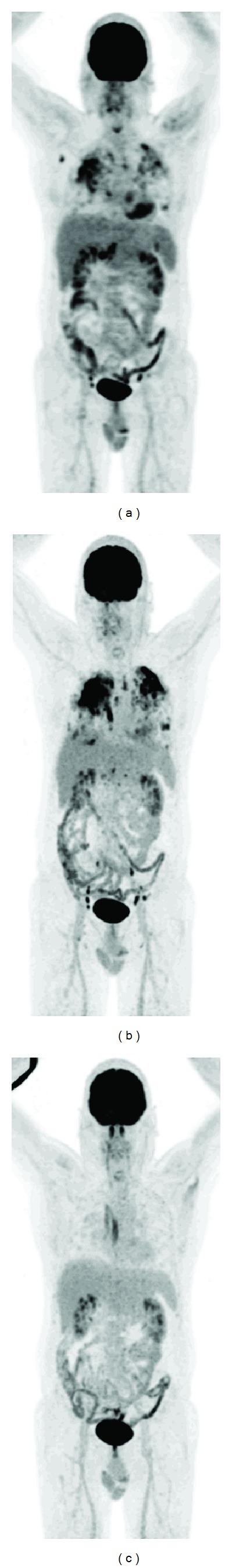
An example of the value of FDG-PET/CT in a patient with sarcoidosis: (a) baseline scan, (b) scan after 3 months of corticosteroid therapy with progression of lung infiltration, and (c) scan after 3 months of treatment with corticosteroids and methotrexate together, resulting in complete remission (the linear uptake is located in a muscle in the back and considered physiological muscle uptake).

Again, there is arguable evidence in the literature: FDG-PET/CT is a valuable imaging tool in assessing treatment efficacy in patients with sarcoidosis and deciding whether to switch to an alternate therapeutic regimen [31, 39–42] (Figure 3). Almost all patients in these studies were treated with corticosteroids, which resulted in a decrease in uptake on FDG-PET but also in clinical and biochemical improvement. 

One study performed FDG-PET/CT before and after 6 cycles of infliximab therapy in 12 patients with refractory sarcoidosis (not reacting on corticosteroid therapy). Clinical improvement was seen in all patients, although minor response in one. FDG-PET improvement or normalization was seen in 11 of the patients with an overall decrease in SUVmax of 55%. However, the patient with a minor clinical response showed a 34% increase in FDG uptake [43]. We may conclude from this study that FDG-PET/CT is not only useful to assess the efficacy of corticosteroid therapy but may also be useful in other therapies, such as infliximab.

## 4. Autoimmune Diseases

### 4.1. Rheumatoid Arthritis

Rheumatoid arthritis (RA) is an autoimmune disease, which is associated with systemic and chronic inflammation of the joints, resulting in synovitis and pannus formation, both leading to increased FDG uptake. Several clinical studies evaluated the role of FDG-PET in patients with RA [44–46]. The degree of FDG uptake in affected joints reflects the disease activity of RA [47] and correlates with clinical parameters, including the disease activity score (DAS), swelling and tenderness, ultrasonography (US) findings for synovitis and synovial thickening, power Doppler studies for neovascularization, ESR, and CRP [48]. FDG-PET was eligible to identify joints with active RA with higher sensitivity than clinical symptoms [47]. The quantification of metabolic changes in joint inflammation with FDG-PET was comparable to volumetric changes visualized with MRI. However, both MRI and FDG-PET were not associated with treatment outcome [48]. 

For therapy evaluation, studies are scarce. Beckers et al. assessed 16 patients with active RA in the knee joint using FDG-PET, dynamic MRI, and US at baseline and four weeks after the initiation of anti-TNF*α* treatment. Significant differences in the MRI and US findings were observed between the FDG-PET positive and FDG-PET negative patients. Changes in the SUV after four weeks were correlated with changes in the MRI parameters, but not with the changes in synovial thickness observed by US [49]. This suggests metabolic changes are preceding morphological changes in patients with RA. 

Goerres et al. used a visual assessment total joint score, that is, the sum of all scores based on FDG uptake intensity between zero and four in 28 joints, in seven patients with active RA before and after infliximab treatment. The reduction of FDG joint uptake in the follow-up scans correlated significantly with clinical evaluation of disease activity [50].

Recently, an association was found between changes in FDG joint uptake between baseline and after two weeks of infliximab treatment and the clinical outcome on long term. Changes in the mean SUV between the baseline scan and the scan after two weeks of treatment correlated significantly with the DAS at 14 and 22 weeks and contributed significantly to the prediction of DAS at these time points. So, early changes in FDG uptake in joints during infliximab treatment may predict clinical outcome [51].

These current collected data together deliver not enough evidence to support the use of FDG-PET for the routine use in patients with RA. In the recently published EULAR recommendation for the use of imaging of the joints in the clinical management of RA, FDG-PET was not recommended as an imaging tool, neither for diagnosis nor for therapy evaluation [52]. To better define the role of FDG-PET in patients with RA, larger patient studies are warranted to understand the clinical usefulness of this technique in this setting.

### 4.2. Inflammatory Bowel Diseases

**Figure 4:**
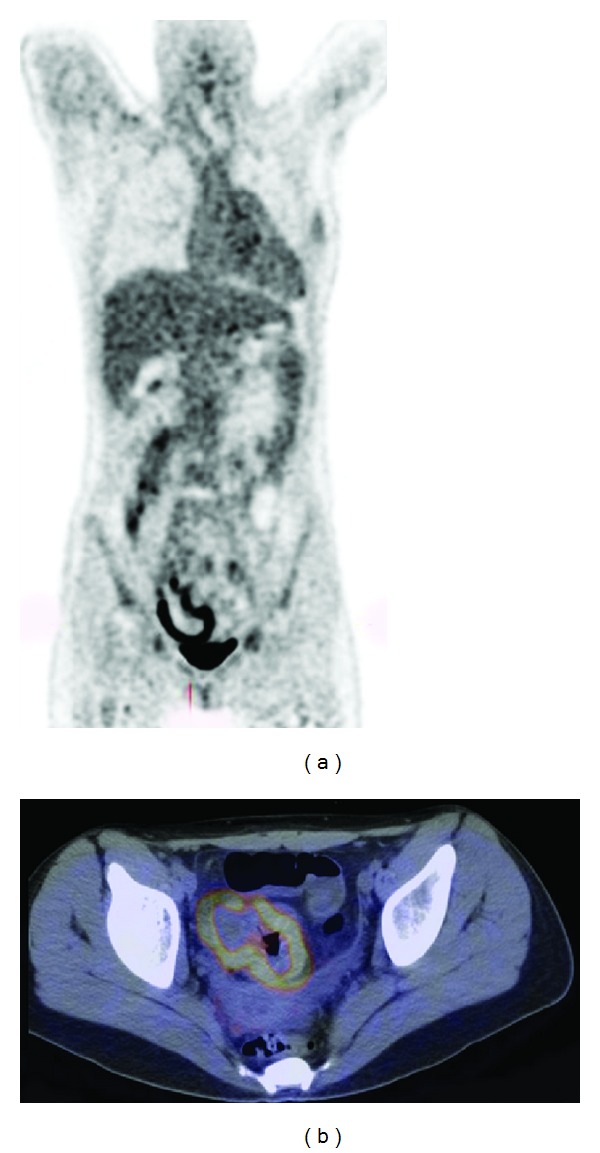
A 14-year-old girl known with Crohn's disease. FDG-PET/CT (left: MIP image of the FDG-PET, right: fused FDG-PET/CT transaxial slice) showed inflammation of the caecum.

Inflammatory bowel diseases (IBD) are represented mainly by ulcerative colitis (UC) and Crohn's disease (CD) and characterized by a chronic, uncontrolled inflammation of the intestinal mucosa. Reported studies in the literature about the use of FDG-PET in IBD—although few in number—concluded that this imaging modality holds potential in evaluating disease activity and providing an objective assessment of the severity of bowel inflammation. Despite these findings, overall not enough literature has been published to support a role for FDG-PET for diagnostic purposes [40, 53]. FDG-PET correlates globally well with clinical activity scores and may be useful when conventional imaging fails to yield a conclusive diagnosis [54]. Despite MRI being the technique of choice in children [55, 56], FDG-PET was found especially suitable for the assessment of IBD in children, where it detected inflamed gut segments with high sensitivity and specificity [57] and could be useful as a noninvasive tool in the followup of children with known chronic IBD, where a yearly invasive colonoscopy is not desirable [58] An example is shown in Figure 4. In another study, the clinical utility of FDG-PET/CT was compared to the standard workup in patients with known or suspected IBD and found very useful, not only in diagnosis but also in therapy management. In this study, unnecessary disease escalation or initiation of IBD therapy was avoided based on the PET/CT results [59].

Despite all these positive results, a major limitation of the use of FDG in IBD is that in a lot of patients gradual physiological uptake in the bowel can be seen, especially in the large bowel, which may create problems in diagnosing IBD in colonic segments. Bowel movements during the scan acquisition can blur the images. Furthermore, diabetic patients who take antidiabetic drugs (e.g., metformin) may show intense uptake in the large bowel. To solve these problems, maybe FDG-PET/CT colonography offers a novel technique for the assessment of extent and activity of IBD. In this technique, the colon is inflated with oral ingestion of polyethylene glycol before acquiring images. In a pilot study in 15 patients, a good correlation was found between PET activity grades after PET/CT colonography and the endoscopic grade of inflammation [60].

Aside from the diagnostic use of FDG, its main utility in IBD can be the early evaluation of treatment success. Indeed FDG represents the whole inflammatory burden of the gut, and an early posttherapy scan (within weeks of beginning of therapy) compared with a pretherapy scan could allow the evaluation of therapy efficacy. To this aim, the only published study is in a small group of 5 patients. FDG-PET/CT was performed in these patients before and after successful medical therapy in patients with moderately active IBD. Five bowel segments were scored on a 0–3 scale (0 = no uptake or uptake lower than liver, 1 = equal to liver, 2 = greater than liver, and 3 = three times liver uptake or higher) for the appropriate FDG-PET assessment. The total score of all segments was 32 before treatment and 14 after treatment. Of 11 pretreatment active segments (score 2 or 3), nine (82%) segments either became inactive or displayed decreased activity, while two showed no change. These findings correlated with clinical symptoms [61]. One major limitation of this study, however, was the time point of the FDG-PET/CT after therapy, this ranged from 77 to 807 days after the baseline scan.

Taken together, all these findings about the use of FDG-PET/CT in IBD demonstrate that currently there are no large patient studies to support the use of this imaging technique for diagnosis and therapy evaluation. This was confirmed in a recently published meta-analysis [62]. However, considering the potential in evaluating disease activity, FDG-PET/CT may have a role to evaluate therapy in IBD in future. 

### 4.3. Autoimmune Thyroiditis

Several forms of autoimmune thyroiditis (AIT) exist, the most important being Riedel's thyroiditis, characterized by a replacement of the normal thyroid parenchyma by a dense fibrosis that invades adjacent structures of the neck and may extend beyond the thyroid capsule, and Hashimoto's thyroiditis (also called lymphocytic thyroiditis), in which the thyroid gland is gradually destroyed by a variety of cell- and antibody-mediated immune processes. AIT can result—on short or longer term—in hypothyroidism.

Normally, uptake of FDG in thyroid tissue is low or absent and unexpected findings in the thyroid gland fall into 2 categories: focal or diffusely increased uptake of FDG. Diffusely increased uptake of FDG in the thyroid is thought to be associated with AIT or hypothyroidism [63] and is mentioned in several case reports [64–66]. In contrast, other authors mention that only 9.5% of PET scans in patients with hypothyroidism as a result of Hashimoto's thyroiditis display diffuse thyroid activity [67].

For the use of FDG-PET as an aid in the followup in Riedel's thyroiditis, Kotilainen et al. describe a 60% decrease in the FDG uptake in the thyroid in the follow-up PET after two weeks of treatment with corticosteroids, indicating that FDG metabolic activity can also be used to assess a patient's response to therapy in Riedel's thyroiditis [68].

One important large retrospective study mentioned different findings. Of 4,732 investigated FDG-PET/CT scans, 138 (2.9%) showed diffuse thyroid uptake. In 47%, a prior diagnosis of hypothyroidism or AIT was found, of whom the majority received thyroxin therapy. In a control group without thyroid uptake, 10% had a prior diagnosis of hypothyroidism and received therapy for that. Of the remaining patients with diffuse thyroid uptake, 32 were examined for thyroid diseases after the findings on the FDG-PET, of which 19 were found to have AIT or hypothyroidism. So, diffusely increased FDG uptake in the thyroid is associated with AIT, but uptake seems not to be affected by hormonal therapy. Furthermore, no correlation was found between SUV and the degree of hypothyroidism [63].

At the moment, there is no special role for FDG-PET/CT, neither in diagnosing AIT nor in evaluating treatment efficacy.

### 4.4. Autoimmune Pancreatitis

Autoimmune pancreatitis (AIP) is a subset of pancreatitis characterized by enlargement of the pancreatic parenchyma and irregular narrowing of the main pancreatic duct, caused by an autoimmune inflammatory process with prominent lymphoplasmacytic infiltration and fibrosis of the pancreas [69]. 

FDG-PET/CT imaging findings in patients with AIP have been explored in a small number of studies. FDG-PET was found to be useful for detecting AIP and associated extra pancreatic autoimmune lesions and also for monitoring disease activity [70–73]. These studies were performed in small numbers of patients, so larger patient studies are definitely warranted.

Another important message is the ability of FDG-PET/CT to differentiate between AIP and pancreatic cancer. Reported sensitivities for pancreatic cancer vary between 72 and 96%, the latter result achieved together with contrast-enhanced CT [74, 75]. For AIP, published studies are too small to calculate sensitivity and specificity. Both conditions normally accumulate FDG, so FDG-PET/CT cannot always discriminate between the two conditions. However, by looking carefully at the pattern of FDG accumulation, accompanying other autoimmune diseases, and the change in FDG uptake after steroid treatment, it may be possible to differentiate between both diseases on the short term. At baseline, FDG-PET shows more diffuse uptake in the pancreas in AIP compared to the more focally located lesions in pancreatic cancer. The detection of other autoimmune diseases, such as uptake in the salivary glands (sclerosing sialadenitis), in the thyroid (AIT) and in the bile ducts (cholangitis), also points towards the diagnosis AIP. Furthermore, a decrease in pancreatic uptake after a short period of steroid therapy (two weeks) may be useful for discriminating AIP from pancreatic cancer [76, 77].

Larger patient studies are necessary to clarify the usefulness of FDG-PET/CT in diagnosis and therapy monitoring. However, when having a diagnostic dilemma between AIP and pancreatic cancer, or when biopsy is not feasible, the FDG uptake pattern and decrease of FDG uptake on the follow-up scan after a short period of therapy, may help to solve this clinical problem.

## 5. Osteomyelitis

The quick identification and precise localization of osteomyelitis (OM) is critical for early initiation of antimicrobial and/or surgical treatment and has a significant impact on patient outcome [78]. FDG-PET/CT has been evaluated in patients with primary OM extensively, has been shown to offer good sensitivity (>95%) and specificities above 87% [79], and was found superior to MRI [80] and other nuclear medicine imaging modalities [81]. However, used in combination with conventional methods, FDG-PET/CT may have limited additional value in the diagnosis of primary OM. In contrast, FDG-PET/CT may play an important role in patients with chronic OM, especially in those patients with previously documented OM and suspected recurrence, or presenting with symptoms of OM for more than six weeks (chronic OM) [3]. In children with suspected OM, dissemination in multiple bones has to be kept in mind, for which FDG-PET/CT would be suitable. However, to avoid radiation exposure, pediatricians tend to perform MRI rather than FDG-PET/CT in cases of suspected OM [82].

For therapy evaluation in patients with OM, the literature results are hopeful. In a recent retrospective study, FDG-PET/CT had a strong impact on the clinical management (initiation or prolongation of antibiotic therapy or recourse to surgical intervention) in 52% of patients with an infection [83]. Gemmel et al. highlighted the clinical role of FDG-PET/CT in the diagnosis of spinal infections, especially in patients with contraindications to MRI, and in evaluation of the postoperative spine [84]. Worth to mention is that in MRI inflammatory changes can be seen long after the disappearance of the infection. For this indication, FDG-PET is probably superior to MRI. In children, FDG-PET/CT was found superior in distinguishing between infection and reparative activity within the musculoskeletal system after treatment for acute OM, and termination of antibiotic treatment for children after acute OM seems justified when laboratory parameters and clinical parameters are normal, and FDG-PET/CT is unsuspicious [85].

Despite these results, major limitations exist for the use of FDG-PET/CT in some infectious bone diseases. In general, in patients with infections or inflammation, the bone marrow, at various levels, can show increased uptake. In prosthetic joint infections (PJI), the problem is the generation of artifacts, characterized by artificial FDG uptake adjacent to prostheses, because of the inherent problem of partial volume mapping and overcorrection. In PJI, white blood cell scintigraphy is still the first choice [86]. Further on, nonspecific FDG uptake may be seen in healing tissues, up to 6 months after surgical intervention [87]. In the diabetic foot, FDG-PET/CT was found to have a low diagnostic accuracy for OM and cannot replace white blood cell scintigraphy [88].

**Figure 5:**
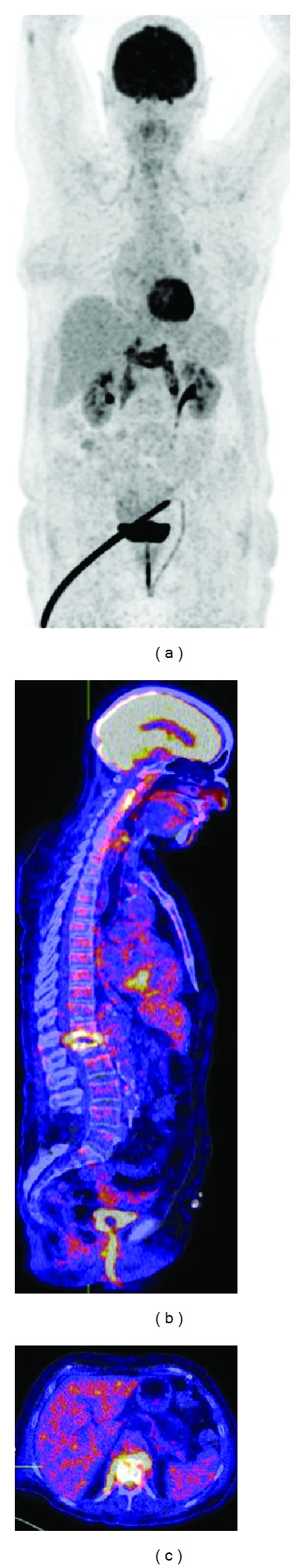
FDG-PET of a patient with spondylodiscitis and involvement of the psoas muscles. (a) Coronal MIP view, (b) fused PET/CT sagittal view, and (c) fused PET/CT transaxial view.

At this moment, evidence-based indications for FDG-PET/CT are primary peripheral bone OM (not post-operative, not in diabetic foot) and suspected spinal infection (spondylodiscitis or vertebral OM, not postoperative; see Figure 5) [89]. In the future, one can expect that FDG-PET/CT will also be used to monitor treatment efficacy, and maybe this technique can provide criteria to decide when the treatment can be stopped safely.

## 6. Fungal Infections

Fungal infections can develop in patients who are taking antibiotics for a long time period (due to an altered balance of microorganisms in the body and an overgrowth of fungus) and in patients with a suppressed immune system, for example, HIV/AIDS, steroid treatment, and chemotherapy. 

In daily practice, FDG-PET/CT has been used already for many years in various fungal infections, such as aspergillosis, candidiasis, histoplasmosis, coccidioidomycosis, cryptococcosis, and *Pneumocystis jiroveci* [90–96]. However, systemic investigations of use of FDG-PET/CT in patients with fungal infections are virtually absent. In the majority, these infections were found by coincidence in patients that were scanned for other reasons. The largest study in diagnosing fungal infections was published by Chamilos et al., who reported their own experiences in 13 patients, together with the results of nine case reports in the literature. Most patients had an underlying malignancy (73%), primarily of hematological origin, and seven were allogeneic hematopoietic stem cell transplant recipients. FDG-PET frequently found occult lesions that were not found with other imaging techniques, and the results helped to determine treatment length in eight of these 16 patients. Overall, FDG-PET was helpful in 60% of the patients [97]. 

The role of FDG-PET in monitoring therapeutic efficacy has also been described in the setting of invasive aspergillosis, chronic disseminated candidiasis, candidal lung abscess following antifungal therapy, and *Pneumocystis carinii* pneumonia, all with good results [98–100]. 

**Figure 6:**
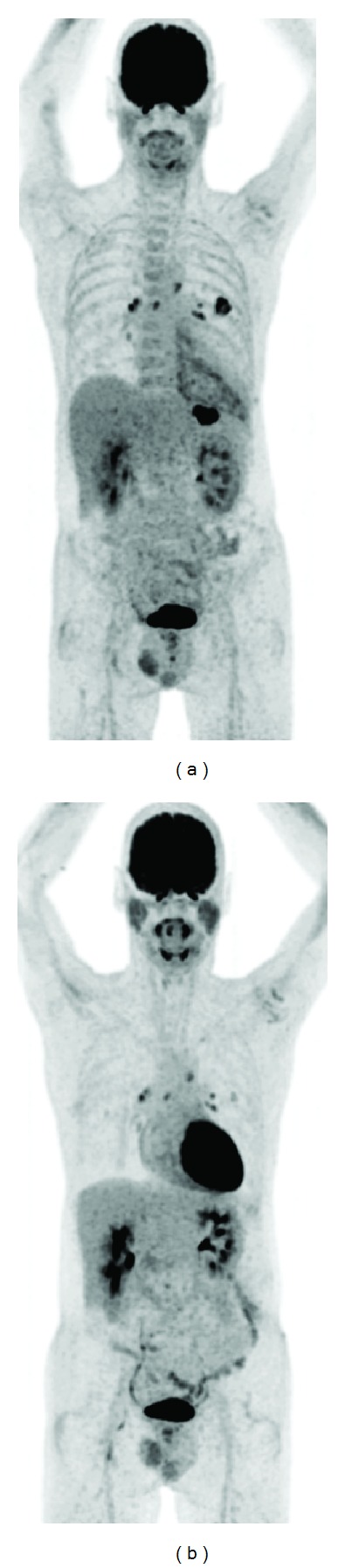
FDG-PET of a neutropenic patient (due to leukemia) with a fungal infection (aspergillosis) before (left image) and during antifungal therapy (right image) resulting in a decrease in FDG uptake in the lung lesions. Because on the last FDG-PET scan (right image) there is still no complete remission, the patient is still treated with antifungal drugs.

In our opinion, FDG-PET/CT offers a unique possibility in the monitoring of therapy efficacy in patients with fungal infections. Antifungal therapy is extensive and must be prolonged for a long time, sometimes even for months. FDG-PET/CT could help to decide whether therapy should be continued, stopped, or switched. An example is given in Figure 6.

## 7. Guidelines

Recently, combined guidelines from the European Association of Nuclear Medicine (EANM) and the Society of Nuclear Medicine and Molecular Imaging (SNMMI) were published for the use of FDG-PET in inflammation and infection [89]. Based on cumulated reported accuracies, >85% of these guidelines state that the major indications for the use of FDG-PET/CT in infection and inflammation are sarcoidosis, peripheral bone osteomyelitis, spondylodiscitis, evaluation of fever of unknown origin, and the primary evaluation of vasculitis. Other well-described applications, however, at this time-point without sufficient evidence-based indications, are the evaluation of potentially infected liver and kidney cysts, suspected infection of intravascular devices, AIDS-associated opportunistic infections, and the assessment of metabolic activity in tuberculosis lesions. For inflammatory bowel diseases, it is unclear if FDG-PET/CT offers advantages over other imaging techniques. The guidelines do not describe the role of FDG-PET/CT for the other described applications in this paper (rheumatoid arthritis, autoimmune thyroiditis, autoimmune pancreatitis, and fungal infections). To our opinion, however not stated in the literature yet, FDG-PET/CT could also have additional value in rheumatoid arthritis and fungal infections. 

## 8. Comparison with Other Tracers

In this paper, we focused only on FDG-PET. To emphasize, one should keep in mind that FDG merely detects glucose metabolism and is therefore not able to discriminate between infection, inflammation, and neoplastic disease. Consequently, there is lack of specificity in some indications, especially in sarcoidosis. Many other, more specific tracers, targeting specific cells and molecules involved in a specific disease, are developed and investigated. These objective biomarkers are used for histological characterization of inflammatory lesions (specific receptor expression), for selection of patients for receptor-targeted therapy (overexpression of a specific receptor), and for therapy response prediction and followup (intensity of receptor expression and its modulation). Other articles are available that provide an overview of all the tracers used for inflammatory and infectious diseases [2]. 

## 9. Conclusions

This paper provides an overview of the use of FDG-PET/CT in various infectious and inflammatory diseases, not only in the setting of diagnosis but also in the evaluation of treatment efficacy (see Table 1). For vasculitis, sarcoidosis, and spondylodiscitis, scientific evidence indicates that FDG-PET/CT is useful for diagnosis and for therapy evaluation. For other diseases, such as inflammatory bowel diseases, rheumatoid arthritis, autoimmune pancreatitis, osteomyelitis, prosthetic joint infection, and fungal infections, a strong evidence is lacking. Only for autoimmune thyroiditis there is lack of evidence, and therefore, FDG-PET/CT should not be used in this setting. 

In any case where FDG can be used for therapy followup, a pretherapy scan is of relevance to compare it with the posttherapy scan.

As it is a repeating problem in nuclear medicine, that no large patient studies are available in the literature to give final answers, we want to stress the importance to start prospective large multicenter studies to provide evidence-based answers. This is not only important in the era of our own imaging field but may also prove to be cost effective as anti-inflammatory therapies are in general expensive.

## Figures and Tables

**Table 1:** Applications of FDG-PET for diagnosis and therapy evaluation in various inflammatory and infectious diseases.

Inflammatory/infectious disease	Evidence for clinical usefulness	Developing evidence for clinical usefulness	Need for multicentre studies	Class of recommendation level of evidence	Comments
Vasculitis					
Diagnosis	+			I-A	Differentiation possible between GCA, TA, and PAN*
Therapy evaluation	+			I-C	Best time point at 3 months
Sarcoidosis					
Diagnosis	+			I-A	High sensitivity, low specificity
Therapy evaluation	+			I-C	To evaluate steroid therapy; maybe also for other therapies
Rheumatoid arthritis					
Diagnosis		+	+	IIa-C	
Therapy evaluation		+	+	IIa-C	
Inflammatory bowel diseases					Especially in children
Diagnosis	+			I-C	
Therapy evaluation		+	+	IIa-C	
Autoimmune thyroiditis					
Diagnosis	−			III-B	
Therapy evaluation	−			III-B	
Autoimmune pancreatitis					
Diagnosis		+	+	IIa-C	Maybe possible to differentiate between AIP and pancreatic cancer
Therapy evaluation		+	+	IIb-C	
Osteomyelitis					
Diagnosis	+			I-A	Only in chronic osteomyelitis
Therapy evaluation		+	+	IIa-C	
Spondylodiscitis					
Diagnosis	+			I-A	
Therapy evaluation		+	+	IIa-C	
Prosthetic joint infections					
Diagnosis	−		+	IIb-C	White blood cell scintigraphy still the first choice
Therapy evaluation	−		+	IIb-C	
Diabetic foot					
Diagnosis	−		+	IIb-C	White blood cell scintigraphy still the first choice
Therapy evaluation	−		+	IIb-C	
Echinococcosis					
Diagnosis	+			I-C	
Therapy evaluation	+			I-C	
Fungal infections					
Diagnosis	+			I-C	
Therapy evaluation		+	+	IIa-C	Maybe helpful to reduce high costs of antifungal therapy

*GCA: giant cell arteritis; TA: Takayasu's arteritis; PAN: polyarteritis nodosa.

## References

[1] Ruf J, Oeser C, Amthauer H (2010). Clinical role of anti-granulocyte MoAb versus radiolabeled white blood cells. *Quarterly Journal of Nuclear Medicine and Molecular Imaging*.

[2] Signore A, Glaudemans AWJM (2011). The molecular imaging approach to image infections and inflammation by nuclear medicine techniques. *Annals of Nuclear Medicine*.

[3] Glaudemans AWJM, Signore A (2010). FDG-PET/CT in infections: the imaging method of choice?. *European Journal of Nuclear Medicine and Molecular Imaging*.

[4] Arend WP, Michel BA, Bloch DA (1990). The American College of Rheumatology 1990 criteria for the classification of Takayasu arteritis. *Arthritis and Rheumatism*.

[5] Hunder GG, Bloch DA, Michel BA (1990). The American College of Rheumatology 1990 criteria for the classification of giant cell arteritis. *Arthritis and Rheumatism*.

[6] Jennette JC, Falk RJ, Andrassy K (1994). Nomenclature of systemic vasculitides: proposal of an international consensus conference. *Arthritis and Rheumatism*.

[7] Zeek PM (1952). Periarteritis nodosa; a critical review. *American Journal of Clinical Pathology*.

[8] Luqmani RA, Suppiah R, Grayson PC, Merkel PA, Watts R (2011). Nomenclature and classification of vasculitis—update on the ACR/EULAR Diagnosis and Classification of Vasculitis Study (DCVAS). *Clinical and Experimental Immunology*.

[9] Bau JL, Ly JQ, Borstad GC, Lusk JD, Seay TM, Beall DP (2003). Giant cell arteritis. *American Journal of Roentgenology*.

[10] Bertagna F, Bosio G, Caobelli F, Motta F, Biasiotto G, Giubbini R (2010). Role of 18F-fl uorodeoxyglucose positron emission tomography/computed tomography for therapy evaluation of patients with large-vessel vasculitis. *Japanese Journal of Radiology*.

[11] Beggs AD, Hain SF (2002). F-18 FDG-positron emission tomographic scanning and Wegener's granulomatosis. *Clinical Nuclear Medicine*.

[12] Morita H, Yokoyama I, Yamada N, Uno K, Nagai R (2004). Usefulness of 18FDG/13N-ammonia PET imaging for evaluation of the cardiac damage in Churg-Strauss syndrome. *European Journal of Nuclear Medicine and Molecular Imaging*.

[13] Yoshibayashi M, Tamaki N, Nishioka K (1991). Regional myocardial perfusion and metabolism assessed by positron emission tomography in children with Kawasaki disease and significance of abnormal Q waves and their disappearance. *American Journal of Cardiology*.

[14] Rauh G, Kamilli I, Gresser U, Landthaler M (1993). Relapsing polychondritis presenting as cutaneous polyarteritis nodosa. *Clinical Investigator*.

[15] Clifford A, Burrell S, Hanly JG (2012). Positron emission tomography/computed tomography for the diagnosis and assessment of giant cell arteritis: when to consider it and why. *The Journal of Rheumatology*.

[16] Zerizer I, Tan K, Khan S (2010). Role of FDG-PET and PET/CT in the diagnosis and management of vasculitis. *European Journal of Radiology*.

[17] Webb M, Chambers A, Al-Nahhas A (2004). The role of 18F-FDG PET in characterising disease activity in Takayasu arteritis. *European Journal of Nuclear Medicine and Molecular Imaging*.

[18] Bleeker-Rovers CP, Bredie SJH, van der Meer JWM, Corstens FHM, Oyen WJG (2003). F-I8-fluorodeoxyglucose positron emmission tomography in diagnosis and follow-up of patients with different types of vasculitis. *Netherlands Journal of Medicine*.

[19] Jaruskova M, Belohlavek O (2006). Role of FDG-PET and PET/CT in the diagnosis of prolonged febrile states. *European Journal of Nuclear Medicine and Molecular Imaging*.

[20] Meller J, Strutz F, Siefker U (2003). Early diagnosis and follow-up of aortitis with [18F]FDG PET and MRI. *European Journal of Nuclear Medicine and Molecular Imaging*.

[21] Walter MA, Melzer RA, Schindler C, Müller-Brand J, Tyndall A, Nitzsche EU (2005). The value of [18F]FDG-PET in the diagnosis of large-vessel vasculitis and the assessment of activity and extent of disease. *European Journal of Nuclear Medicine and Molecular Imaging*.

[22] Blockmans D, De Ceuninck L, Vanderschueren S, Knockaert D, Mortelmans L, Bobbaers H (2006). Repetitive18F-fluorodeoxyglucose positron emission tomography in giant cell arteritis: a prospective study of 35 patients. *Arthritis Care and Research*.

[23] Henes JC, Müller M, Pfannenberg C, Kanz L, Kötter I (2011). Cyclophosphamide for large-vessel vasculitis: assessment of response by PET/CT. *Clinical and Experimental Rheumatology*.

[24] Fuchs M, Briel M, Daikeler T (2012). The impact of18F-FDG PET on the management of patients with suspected large vessel vasculitis. *European Journal of Nuclear Medicine and Molecular Imaging*.

[25] Scadding JG (1961). Prognosis of intrathoracic sarcoidosis in England. A review of 136 cases after five years' observation. *British Medical Journal*.

[26] Nunes H, Brillet P-Y, Valeyre D, Brauner MW, Wells AU (2007). Imaging in sarcoidosis. *Seminars in Respiratory and Critical Care Medicine*.

[27] Hansell DM (2001). High-resolution CT of diffuse lung disease: value and limitations. *Radiologic Clinics of North America*.

[28] Lee KS, Primack SL, Staples CA, Mayo JR, Aldrich JE, Müller NL (1994). Chronic infiltrative lung disease: comparison of diagnostic accuracies of radiography and low- and conventional-dose thin-section CT. *Radiology*.

[29] Mana J, Salazar A, Manresa F (1994). Clinical factors predicting persistence of activity in sarcoidosis: a multivariate analysis of 193 cases. *Respiration*.

[30] Takada K, Ina Y, Noda M, Sato T, Yamamoto M, Morishita M (1993). The clinical course and prognosis of patients with severe, moderate or mild sarcoidosis. *Journal of Clinical Epidemiology*.

[31] Teirstein AS, Machac J, Almeida O, Lu P, Padilla ML, Iannuzzi MC (2007). Results of 188 whole-body fluorodeoxyglucose positron emission tomography scans in 137 patients with sarcoidosis. *Chest*.

[32] Brancato SC, Arrighi JA (2011). Fasting FDG PET compared to MPI SPECT in cardiac sarcoidosis. *Journal of Nuclear Cardiology*.

[33] Aide N, Benayoun M, Kerrou K, Khalil A, Cadranel J, Talbot JN (2007). Impact of [18F]-fluorodeoxyglucose ([18F]-FDG) imaging in sarcoidosis: unsuspected neurosarcoidosis discovered by [18F]-FDG PET and early metabolic response to corticosteroid therapy. *The British Journal of Radiology*.

[34] Keijsers RG, Grutters JC, Thomeer M (2011). Imaging the inflammatory activity of sarcoidosis: sensitivity and inter observer agreement of67Ga imaging and18F-FDG PET. *Quarterly Journal of Nuclear Medicine and Molecular Imaging*.

[35] Prager E, Wehrschuetz M, Bisail B (2008). Comparison of 18F-FDG and 67Ga-citrate in sarcoidosis imaging. *NuklearMedizin*.

[36] Keijsers RG, Grutters JC, van Velzen-Blad H, van den Bosch JM, Oyen WJ, Verzijlbergen FJ (2010). 18F-FDG PET patterns and BAL cell profiles in pulmonary sarcoidosis. *European Journal of Nuclear Medicine and Molecular Imaging*.

[37] Keijsers RG, Verzijlbergen FJ, van den Bosch JM (2011). 18F-FDG PET as a predictor of pulmonary function in sarcoidosis. *Sarcoidosis Vasculitis and Diffuse Lung Diseases*.

[38] Soussan M, Augier A, Brillet PY, Weinmann P, Valeyre D (2013). Functional imaging in extrapulmonary sarcoidosis: FDG-PET/CT and MR features. *Clinical Nuclear Medicine*.

[39] Aide N, Allouache D, Ollivier Y, de Raucourt S, Switsers O, Bardet S (2009). Early 2′-Deoxy-2′-[18F]Fluoro-D-Glucose PET metabolic response after corticosteroid therapy to differentiate cancer from sarcoidosis and sarcoid-like lesions. *Molecular Imaging and Biology*.

[40] Basu S, Zhuang H, Torigian DA, Rosenbaum J, Chen W, Alavi A (2009). Functional imaging of inflammatory diseases using nuclear medicine techniques. *Seminars in Nuclear Medicine*.

[41] Braun JJ, Kessler R, Constantinesco A, Imperiale A (2008). 18F-FDG PET/CT in sarcoidosis management: review and report of 20 cases. *European Journal of Nuclear Medicine and Molecular Imaging*.

[42] Imperiale A, Riehm S, Veillon F, Namer I-J, Braun J-J (2011). FDG PET coregistered to MRI for diagnosis and monitoring of therapeutic response in aggressive phenotype of sarcoidosis. *European Journal of Nuclear Medicine and Molecular Imaging*.

[43] Keijsers RGM, Verzijlbergen JF, van Diepen DM, van den Bosch JMM, Grutters JC (2008). 18F-FDG PET in sarcoidosis: an observational study in 12 patients treated with infliximab. *Sarcoidosis Vasculitis and Diffuse Lung Diseases*.

[44] Hashefi M, Curiel R (2011). Future and upcoming non-neoplastic applications of PET/CT imaging. *Annals of the New York Academy of Sciences*.

[45] Kubota K, Ito K, Morooka M (2009). Whole-body FDG-PET/CT on rheumatoid arthritis of large joints. *Annals of Nuclear Medicine*.

[46] McBride HJ (2010). Nuclear imaging of autoimmunity: focus on IBD and RA. *Autoimmunity*.

[47] Kubota K, Ito K, Morooka M (2011). FDG PET for rheumatoid arthritis: basic considerations and whole-body PET/CT. *Annals of the New York Academy of Sciences*.

[48] Palmer WE, Rosenthal DI, Schoenberg OI (1995). Quantification of inflammation in the wrist with gadolinium-enhanced MR imaging and pet with 2-[F-18]-fluoro-2-deoxy-D-glucose. *Radiology*.

[49] Beckers C, Ribbens C, André B (2004). Assessment of disease activity in rheumatoid arthritis with 18F-FDG PET. *Journal of Nuclear Medicine*.

[50] Goerres GW, Forster A, Uebelhart D (2006). F-18 FDG whole-body PET for the assessment of disease activity in patients with rheumatoid arthritis. *Clinical Nuclear Medicine*.

[51] Elzinga EH, van der Laken CJ, Comans EFI (2011). 18F-FDG PET as a tool to predict the clinical outcome of infliximab treatment of rheumatoid arthritis: an explorative study. *Journal of Nuclear Medicine*.

[52] Colebatch AN, Edwards CJ, Ostergaard M (2013). EULAR recommendations for the use of imaging of the joints in the clinical management of rheumatoid arthritis. *Annals of the Rheumatic Diseases*.

[53] Chandler MB, Zeddun SM, Borum ML (2011). The role of positron emission tomography in the evaluation of inflammatory bowel disease. *Annals of the New York Academy of Sciences*.

[54] Glaudemans AWJM, Maccioni F, Mansi L, Dierckx RAJO, Signore A (2010). Imaging of cell trafficking in Crohn’s disease. *Journal of Cellular Physiology*.

[55] de Bie CI, Buderus S, Sandhu BK (2012). Diagnostic workup of paediatric patients with inflammatory bowel disease in Europe: results of a 5-year audit of the EUROKIDS registry. *Journal of Pediatric Gastroenterology and Nutrition*.

[56] Sauer CG, Kugathasan S, Martin DR, Applegate KE (2011). Medical radiation exposure in children with inflammatory bowel disease estimates high cumulative doses. *Inflammatory Bowel Diseases*.

[57] Däbritz J, Jasper N, Loeffler M, Weckesser M, Foell D (2011). Noninvasive assessment of pediatric inflammatory bowel disease with 18F-fluorodeoxyglucose-positron emission tomography and computed tomography. *European Journal of Gastroenterology and Hepatology*.

[58] Löffler M, Weckesser M, Franzius C, Schober O, Zimmer K-P (2006). High diagnostic value of 18F-FDG-PET in pediatric patients with chronic inflammatory bowel disease. *Annals of the New York Academy of Sciences*.

[59] Lapp RT, Spier BJ, Perlman SB, Jaskowiak CJ, Reichelderfer M (2011). Clinical utility of positron emission tomography/computed tomography in inflammatory bowel disease. *Molecular Imaging and Biology*.

[60] Das CJ, Makharia GK, Kumar R (2010). PET/CT colonography: a novel non-invasive technique for assessment of extent and activity of ulcerative colitis. *European Journal of Nuclear Medicine and Molecular Imaging*.

[61] Spier BJ, Perlman SB, Jaskowiak CJ, Reichelderfer M (2010). PET/CT in the evaluation of inflammatory bowel disease: studies in patients before and after treatment. *Molecular Imaging and Biology*.

[62] Treglia G, Quartuccio N, Sadeghi R (2013). Diagnostic performance of fluorine-18-fluorodeoxyglucose positron emission tomography in patients with chronic inflammatory bowel disease: a systematic review and a meta-analysis. *Journal of Crohn's and Colitis*.

[63] Karantanis D, Bogsrud TV, Wiseman GA (2007). Clinical significance of diffusely increased 18F-FDG uptake in the thyroid gland. *Journal of Nuclear Medicine*.

[64] Slman R, Monpeyssen H, Desarnaud S (2011). Ultrasound, elastography, and fluorodeoxyglucose positron emission tomography/computed tomography imaging in riedel’s thyroiditis: report of two cases. *Thyroid*.

[65] Song YS, Jang SJ, Chung J-K, Lee DS (2009). F-18 fluorodeoxyglucose (FDG) positron emission tomography (PET) and Tc-99m pertechnate scan findings of a patient with unilateral subacute thyroiditis. *Clinical Nuclear Medicine*.

[66] Yeo SH, Lee SK, Hwang I, Ahn EJ (2011). Subacute thyroiditis presenting as a focal lesion on [18F] fluorodeoxyglucose whole-body positron-emission tomography/CT. *American Journal of Neuroradiology*.

[67] Rothman IN, Middleton L, Stack BC, Bartel T, Riggs AT, Bodenner DL (2011). Incidence of diffuse FDG uptake in the thyroid of patients with hypothyroidism. *European Archives of Oto-Rhino-Laryngology*.

[68] Kotilainen P, Airas L, Kojo T (2004). Positron emission tomography as an aid in the diagnosis and follow-up of Riedel’s thyroiditis. *European Journal of Internal Medicine*.

[69] Okazaki K, Uchida K, Fukui T (2008). Recent advances in autoimmune pancreatitis: concept, diagnosis, and pathogenesis. *Journal of Gastroenterology*.

[70] Kamisawa T, Takum K, Anjiki H (2010). FDG-PET/CT findings of autoimmune pancreatitis. *Hepato-Gastroenterology*.

[71] Matsubayashi H, Furukawa H, Maeda A (2009). Usefulness of positron emission tomography in the evaluation of distribution and activity of systemic lesions associated with autoimmune pancreatitis. *Pancreatology*.

[72] Nakajo M, Jinnouchi S, Fukukura Y, Tanabe H, Tateno R, Nakajo M (2007). The efficacy of whole-body FDG-PET or PET/CT for autoimmune pancreatitis and associated extrapancreatic autoimmune lesions. *European Journal of Nuclear Medicine and Molecular Imaging*.

[73] Nakajo M, Jinnouchi S, Noguchi M (2007). FDG PET and PET/CT monitoring of autoimmune pancreatitis associated with extrapancreatic autoimmune disease. *Clinical Nuclear Medicine*.

[74] Buchs NC, Bühler L, Bucher P (2011). Value of contrast-enhanced18F-fluorodeoxyglucose positron emission tomography/computed tomography in detection and presurgical assessment of pancreatic cancer: a prospective study. *Journal of Gastroenterology and Hepatology*.

[75] Lee SM, Kim T-S, Lee JW, Kim S-K, Park S-J, Han S-S (2011). Improved prognostic value of standardized uptake value corrected for blood glucose level in pancreatic cancer using F-18 FDG PET. *Clinical Nuclear Medicine*.

[76] Tae YL, Kim M-H, Do HP (2009). Utility of18F-FDG PET/CT for differentiation of autoimmune pancreatitis with atypical pancreatic imaging findings from pancreatic cancer. *American Journal of Roentgenology*.

[77] Shigekawa M, Yamao K, Sawaki A (2010). Is18F-fluorodeoxyglucose positron emission tomography meaningful for estimating the efficacy of corticosteroid therapy in patients with autoimmune pancreatitis?. *Journal of Hepato-Biliary-Pancreatic Sciences*.

[78] Concia E, Prandini N, Massari L (2006). Osteomyelitis: clinical update for practical guidelines. *Nuclear Medicine Communications*.

[79] Gotthardt M, Bleeker-Rovers CP, Boerman OC, Oyen WJG (2010). Imaging of inflammation by PET, conventional scintigraphy, and other imaging techniques. *Journal of Nuclear Medicine*.

[80] Gratz S, Dörner J, Fischer U (2002). 18F-FDG hybrid PET in patients with suspected spondylitis. *European Journal of Nuclear Medicine*.

[81] Wang G-L, Zhao K, Liu Z-F, Dong M-J, Yang S-Y (2011). A meta-analysis of fluorodeoxyglucose-positron emission tomography versus scintigraphy in the evaluation of suspected osteomyelitis. *Nuclear Medicine Communications*.

[82] Darge K, Jaramillo D, Siegel MJ (2008). Whole-body MRI in children: current status and future applications. *European Journal of Radiology*.

[83] Ito K, Kubota K, Morooka M, Hasuo K, Kuroki H, Mimori A (2010). Clinical impact of 18F-FDG PET/CT on the management and diagnosis of infectious spondylitis. *Nuclear Medicine Communications*.

[84] Gemmel F, Rijk PC, Collins JMP, Parlevliet T, Stumpe KD, Palestro CJ (2010). Expanding role of 18F-fluoro-d-deoxyglucose PET and PET/CT in spinal infections. *European Spine Journal*.

[85] Warmann SW, Dittmann H, Seitz G, Bares R, Fuchs J, Schäfer JF (2011). Follow-up of acute osteomyelitis in children: the possible role of PET/CT in selected cases. *Journal of Pediatric Surgery*.

[86] Leone S, Borrè S, Monforte AD (2010). Consensus document on controversial issues in the diagnosis and treatment of prosthetic joint infections. *International Journal of Infectious Diseases*.

[87] Jones-Jackson L, Walker R, Purnell G (2005). Early detection of bone infection and differentiation from post-surgical inflammation using 2-deoxy-2-[18F]-fluoro-D-glucose positron emission tomography (FDG-PET) in an animal model. *Journal of Orthopaedic Research*.

[88] Familiari D, Glaudemans AWJM, Vitale V (2011). Can sequential18F-FDG PET/CT replace WBC imaging in the diabetic foot?. *Journal of Nuclear Medicine*.

[89] Jamar F, Buscombe J, Chiti A (2013). EANM/SNMMI guideline for 18F-FDG use in inflammation and infection. *Journal of Nuclear Medicine*.

[90] Bleeker-Rovers CP, Warris A, Drenth JPH, Corstens FHM, Oyen WJG, Kullberg B-J (2005). Diagnosis of Candida lung abscesses by 18F-fluorodeoxyglucose positron emission tomography. *Clinical Microbiology and Infection*.

[91] Huang C-J, You D-L, Lee P-I (2009). Characteristics of integrated18F-FDG PET/CT in pulmonary cryptococcosis. *Acta Radiologica*.

[92] Igai H, Gotoh M, Yokomise H (2006). Computed tomography (CT) and positron emission tomography with [18F]fluoro-2-deoxy-d-glucose (FDG-PET) images of pulmonary cryptococcosis mimicking lung cancer. *European Journal of Cardio-Thoracic Surgery*.

[93] Kobayashi E, Iwamiya T, Masaki H (2009). Postoperative abdominal aspergilloma mimicking cervical cancer recurrence and diagnostic imaging, including18F-fluorodeoxyglucose positron emission tomography, with false-positive findings. *Journal of Obstetrics and Gynaecology Research*.

[94] Salhab KF, Baram D, Bilfinger TV (2006). Growing PET positive nodule in a patient with histoplasmosis: case report. *Journal of Cardiothoracic Surgery*.

[95] Sojan SM, Chew G (2005). Pneumocystis carinii Pneumonia on F-18 FDG PET. *Clinical Nuclear Medicine*.

[96] Teyton P, Baillet G, Hindié E (2009). Hepatosplenic candidiasis imaged with F-18 FDG PET/CT. *Clinical Nuclear Medicine*.

[97] Chamilos G, Macapinlac HA, Kontoyiannis DP (2008). The use of 18F-fluorodeoxyglucose positron emission tomography for the diagnosis and management of invasive mould infections. *Medical Mycology*.

[98] Basu S, Saboury B, Werner T, Alavi A (2011). Clinical utility of FDG-PET and PET/CT in non-malignant thoracic disorders. *Molecular Imaging and Biology*.

[99] Franzius C, Biermann M, HüLskamp G (2001). Therapy monitoring in aspergillosis using F-18 FDG positron emission tomography. *Clinical Nuclear Medicine*.

[100] Xu B, Shi P, Wu H, Guo X, Wang Q, Zhou S (2010). Utility of FDG PET/CT in guiding antifungal therapy in acute leukemia patients with chronic disseminated candidiasis. *Clinical Nuclear Medicine*.

